# Molecular Insulin Actions Are Sexually Dimorphic in Lipid Metabolism

**DOI:** 10.3389/fendo.2021.690484

**Published:** 2021-06-18

**Authors:** Rosa Isela Ortiz-Huidobro, Myrian Velasco, Carlos Larqué, Rene Escalona, Marcia Hiriart

**Affiliations:** ^1^ Neurosciences Division, Department of Cognitive Neuroscience, Instituto de Fisiología Celular, Universidad Nacional Autónoma de México, Ciudad de México, Mexico; ^2^ Department of Embryology and Genetics, Facultad de Medicina, Universidad Nacional Autónoma de México, Ciudad de México, Mexico

**Keywords:** insulin signaling pathway, sexual dimorphism, lipid metabolism, insulin resistance, metabolic dysfunction, obesity, estrogens, testosterone

## Abstract

The increment in energy-dense food and low physical activity has contributed to the current obesity pandemic, which is more prevalent in women than in men. Insulin is an anabolic hormone that regulates the metabolism of lipids, carbohydrates, and proteins in adipose tissue, liver, and skeletal muscle. During obesity, nutrient storage capacity is dysregulated due to a reduced insulin action on its target organs, producing insulin resistance, an early marker of metabolic dysfunction. Insulin resistance in adipose tissue is central in metabolic diseases due to the critical role that this tissue plays in energy homeostasis. We focused on sexual dimorphism on the molecular mechanisms of insulin actions and their relationship with the physiology and pathophysiology of adipose tissue. Until recently, most of the physiological and pharmacological studies were done in males without considering sexual dimorphism, which is relevant. There is ample clinical and epidemiological evidence of its contribution to the establishment and progression of metabolic diseases. Sexual dimorphism is a critical and often overlooked factor that should be considered in design of sex-targeted therapeutic strategies and public health policies to address obesity and diabetes.

## Introduction

One of the complex problems in modern society is that the availability of energy-dense food has led to an imbalance between the calories consumed and burned. A sedentary lifestyle has induced this imbalance. These factors undoubtedly have contributed to the recent obesity and diabetes pandemic and the increase in cardiovascular diseases. On the other hand, sexual dimorphism relies on morphological and biological disparities that influence physiological or pathophysiological processes in males and females (1).

Epidemiological data reveal differences in the incidence and prevalence among men and women of overweight, obesity, metabolic syndrome, and as a consequence, type-2 diabetes mellitus (T2DM) and cardiovascular diseases (CVDs). Furthermore, clinical and experimental evidence of sex-specific components in the development of these diseases supports these facts (2). However, the genetics and molecular mechanisms underlying these different responses are not entirely clear.

Adipose tissue plays a central role in regulating insulin sensitivity and glucose homeostasis (3, 4). Insulin resistance is a diminished ability of cells to respond to the physiological actions of insulin. It is a well-known risk factor the establishment and progression of T2DM and CVDs (5). This work aims to analyze the sex-specific mechanisms of the regulation of adipose tissue function by insulin to gain a better insight into the molecular mechanisms associated with developing metabolic diseases.

We will first address the insulin actions and signaling pathway and sexual dimorphism of insulin actions on gonadal development. Next, we will review the current knowledge of sex differences in adipose tissue physiology, highlighting the role of estrogens in lipid metabolism and their relationship with insulin secretion by pancreatic beta-cells. Finally, we will explore adipose dysfunction in both sexes, specifically in obesity, inflammation, and dyslipidemia. We will center on some proteins of insulin signaling identified to show sexual dimorphism.

## Metabolic Insulin Actions and Signaling Pathway

The metabolic actions of insulin are of great importance for energy homeostasis in the organism´s function. All the tissues express insulin receptors; however, metabolic actions of insulin are more understood in the liver, adipose tissue, and skeletal muscle. In these organs, insulin regulates glucose homeostasis through the balance between storage and mobilization of energy reserves during the feeding and fasting states (6).

Insulin promotes protein and lipid synthesis and the storage of glucose as glycogen in muscles and the liver. In skeletal muscle and adipose tissue, insulin regulates glucose uptake through of the translocation of glucose transporter type 4 (GLUT4) from an intracellular pool to the plasma membrane. Besides, in adipose tissue, insulin promotes lipogenesis and inhibits lipolysis. In the liver, insulin also suppresses the production of glucose and promotes *de novo* fatty acid synthesis. Thus, insulin regulates the concentration of glucose and fatty acids in circulation. In these and other tissues, insulin also regulates gene expression and division, survival, and cell growth (7).

In the feeding state, nutrients enter the intestine and then the portal system, reaching the pancreatic islets, where beta-cells reside. In response, these cells secrete insulin that is transported toward other organs (8).

In its target tissues, insulin binds to the extracellular alpha subunits of the insulin receptor (IR). It produces conformational changes of beta subunits of receptor in the cytoplasmatic domain, promoting the subunits´ transphosphorylation. The tyrosine kinase activity starts a cascade of phosphorylations that transduces the insulin signal within cells.

**Figure 1:**
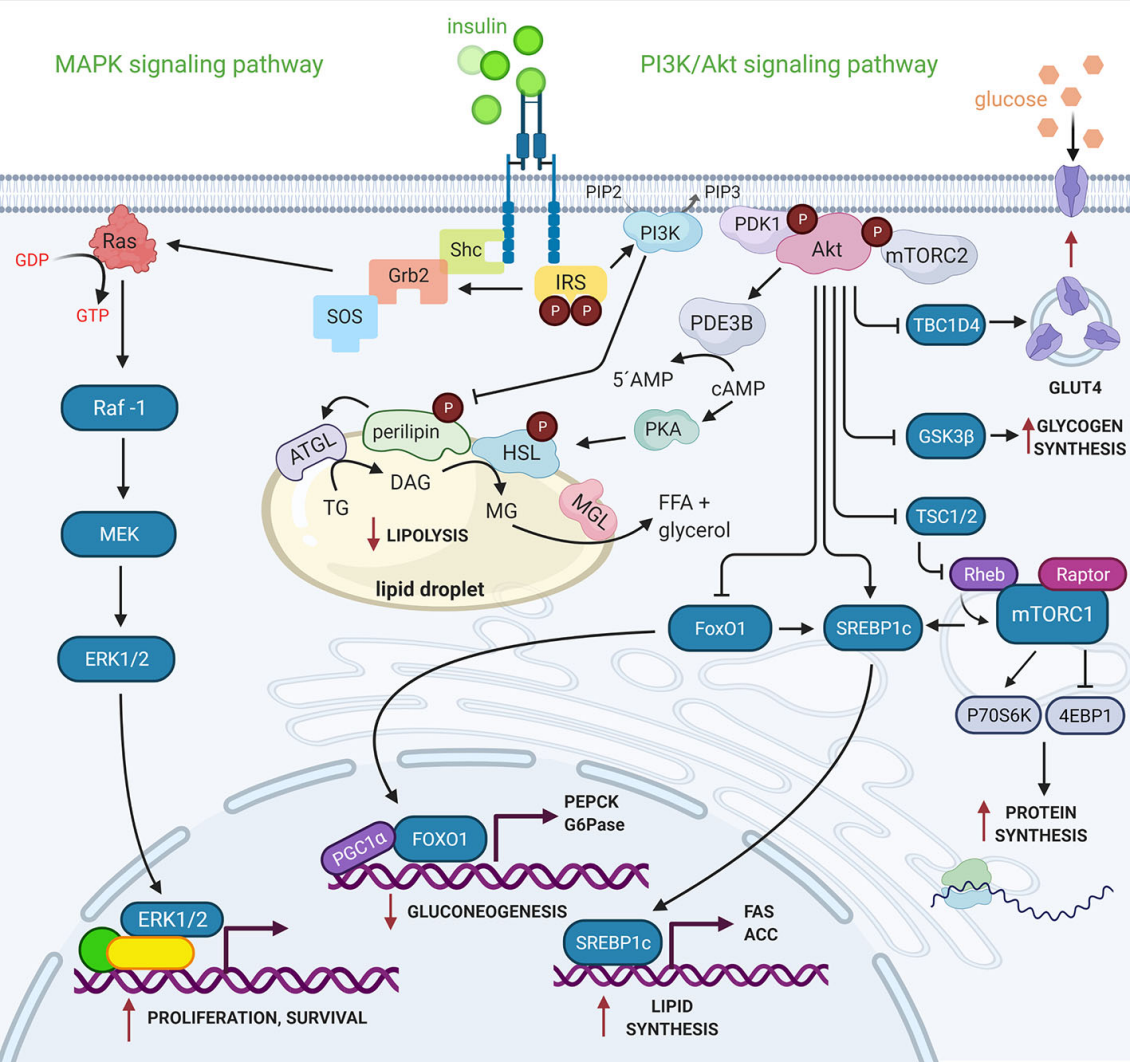
PI3K/Akt and MAPK signaling pathways activated by insulin. Active IR transduces insulin signal to effector proteins downstream, as the IRS proteins family provides specific scaffolding sites that activate other kinases such as PI3K. The catalytic subunit (p110) of PI3K interacts with its substrate, PI (4,5)P2 in the cell membrane, generating PI (3,4,5)P3, which serves as the binding site of the PDK1 and mTORC2 kinases. The mTORC2 protein complex activates Akt, inducing the first phosphorylation in Ser^473^ followed by another PDK1-induced phosphorylation in Thr^308^. Akt regulates the metabolic effects of insulin through phosphorylation of a wide variety of substrates. On the other hand, IR besides phosphorylating to IRS, also phosphorylates to Shc protein, both independently can activate the Grb2 protein that through the SOS nucleotide exchanger activates to GTPase Ras, which transduces the signal to the MAP kinases (Raf-1, MEK y ERK 1/2). ERK1/2 activates transcription factors and nuclear proteins. Insulin thus modulates gene expression and cell growth (7, 9). See attached list of abbreviations. Created with BioRender.com.

Insulin activates two main signaling pathways: the PI3K/Akt signaling pathway, which directs insulin´s primary metabolic functions, and the MAPK signaling pathway, which regulates the mitogenic effects of insulin (9) (**Figure 1**).

## Starting in the Intrauterine Period, Insulin Actions Differ in Males and Females

Sexual dimorphism arises in part due to the development of an ovary and a testis from a bipotential gonad. In mammals, gonad development drives the differential production and secretion of steroid hormones. In most cases, the physiological and pathological differences observed between males and females result from this sex-specific hormone secretion pattern.

It is worth mentioning that there is sexual dimorphism in early development stages, thus demonstrating the role of a genetic/chromosomic component (10). For example, in mice (11) and humans (12) the male and female embryos show different growth and metabolic rates before implantation. Furthermore, there are differences in insulin actions during different developmental stages in male and female gonads.

The role of insulin in the testis begins even before birth. Testis development in mice requires the presence of the insulin receptor family (13); XY mutants lacking the *Insr/Igfr* receptors in the gonad display a male-to-female sex reversal. Their gonads have ovarian morphology; in addition, they do not have Sertoli and Leydig cell differentiation. Additionally, a recent study has shown that mice with a deletion of *Insr/Igfr* in steroidogenic cells displayed smaller testes, reduced sperm concentration, and lower serum testosterone levels (14). However, the gonads still display a male phenotype (i.e., seminiferous cords were present), albeit with a significant reduction in Leydig cell numbers in the adult testis. The role of insulin in Sertoli cells seems to be similar to that observed for Leydig cells. Adult mice with a Sertoli cell-specific deletion of *Insr/Igf1r* have fewer Sertoli cells, smaller testis, altered morphology of the seminiferous tubes, and a reduced sperm count (15). Supporting the role of insulin in testicular development, mice lacking IRS2, besides having impaired glucose homeostasis, have smaller testes and reduced fertility (16).

Reproduction and nutrition are tightly interrelated; thus, insulin plays an essential role in male and female reproduction. The human ovary expresses all but two glucose transporters (GLUT2 and GLUT7), and glucose is their primary energy source (17); also for the ovine ovary (18).

Insulin-dependent glucose uptake by the seminiferous cords in the rat testis occurs in a PI3K/Akt signaling-dependent manner (19). Reproductive impairment is frequently associated to alterations in insulin signaling.

Reduced glucose intake in the cumulus cells from diabetic mothers has been associated with low oocyte quality (20). Additionally, obese women, often insulin resistant, have a higher incidence of infertility (21). Furthermore, insulin resistance is associated with several reproductive anomalies, such as polycystic ovarian syndrome (PCOS) (22). In addition, women with metabolic syndrome and insulin resistance require a longer time to become pregnant independently of obesity (23). Furthermore, in a small case-control study, seminal fluid from obese men contained a higher insulin concentration than non-obese men (24); correlated with a decreased sperm quality. These results indicate that regardless of sex, high plasma insulin concentration and reduced insulin sensitivity are linked to reduced fertility.

The human growing follicles express insulin receptors (25). Insulin modulates the steroidogenic actions of granulosa cells. For example, insulin promotes estradiol secretion and aromatase activity in bovine granulosa cells stimulated with FSH (26). One explanation for this synergy is that FSH regulates the genes for sterol synthesis *via* the FoxO1 transcription factor (27), a signaling node shared with insulin.

Insulin´s synergistic effect over gonadotropins is also observed in LH-treated human theca cells by promoting testosterone and androstenedione secretion (28). Insulin also promotes theca cell proliferation in an mTOR-dependent manner (29).

In contrast, insulin does not appear to have a metabolic role in the oocyte despite the expression of insulin receptors. The oocyte depends on cumulus cells to obtain energy substrates such as pyruvate *via* gap junctions (20). One possible role of insulin signaling in the oocyte could be the regulation of meiotic progression. Oocytes from diabetic mice have a delayed maturation *via* deregulation of AMPK activity (30). In addition, they display an increased frequency of abnormal meiotic spindles (31).

In contrast to the ovary, the role of insulin over the production of steroid hormones in the testis seems to be an inhibitory one. In an *in vitro* assay, insulin reduced progesterone and testosterone in Leydig cells *via* Akt. The same study compared these results to obese mice fed with a high-fat diet (HFD). These mice displayed a similar reduction in steroid hormones and increased in p-Akt in the testis (32). In addition, there is clinical evidence that men with insulin resistance have lower plasma testosterone (33).

In conclusion, insulin has a differential role in the gonads; furthermore, insulin and sex hormone´s actions are interrelated. Insulin is capable of modulating sex hormone synthesis and effects. In turn, sex hormones affect tissue response to insulin. Thus, insulin actions vary between males in females in part due to their specific hormonal profile. In subsequent sections, we will discuss how this hormonal profile affects insulin sensitivity in normal and pathological settings.

## Sexual Dimorphism in Adipose Tissue and Lipid Metabolism

As previously discussed, sexual dimorphism is established during the early embryonic and fetal stages of development although, it is more evident during the post-puberty phase. At birth, males and females have a similar fat mass; however, males show more lean mass and are taller than females. Those differences are less evident during childhood. Nevertheless, during puberty, both sexes undergo marked significant changes due to the effects of sex hormones. In general, adult men have a substantial total lean and mineral mass and a lower fat mass than women (34).

Adipose tissue is an important endocrine and metabolically very active organ; its functions include mechanical protection, thermogenesis, storage and release of energy reserves, regulation of immune response, and secretion of adipokines. The amount of plasma adipokines indicates the metabolic status, and they directly act over different organs (35).

In addition, there are differences in adipose tissue distribution between sexes. Women have extensive subcutaneous adipose tissue (femoral and gluteal depots, so-called gynoid phenotype). In contrast, men accumulate fat mainly in visceral adipose tissue (so-called android phenotype) (36).

Furthermore, visceral and subcutaneous adipose tissue depots have different metabolic activity (35). Therefore, it is comprehensible that sex differences in adipose tissue distribution determine the differential metabolic phenotype between males and females. These differences in the metabolic profile include insulin sensitivity, free fatty acid (FFA) release, and adipokines production (37).

There is a clear association between visceral adiposity and reduced insulin sensitivity; conversely, subcutaneous adiposity associates with increased insulin sensitivity (38). In mice, intra-abdominal, perigonadal, and subcutaneous adipocytes display an increased lipogenesis in females compared to males. Moreover, stimulation of female visceral adipocytes with a low insulin concentration, increased the phosphorylated Akt and ERK protein levels (39). This differential activity could explain why women are more sensitive to insulin; despite increased adiposity compared to men.

**Figure 2:**
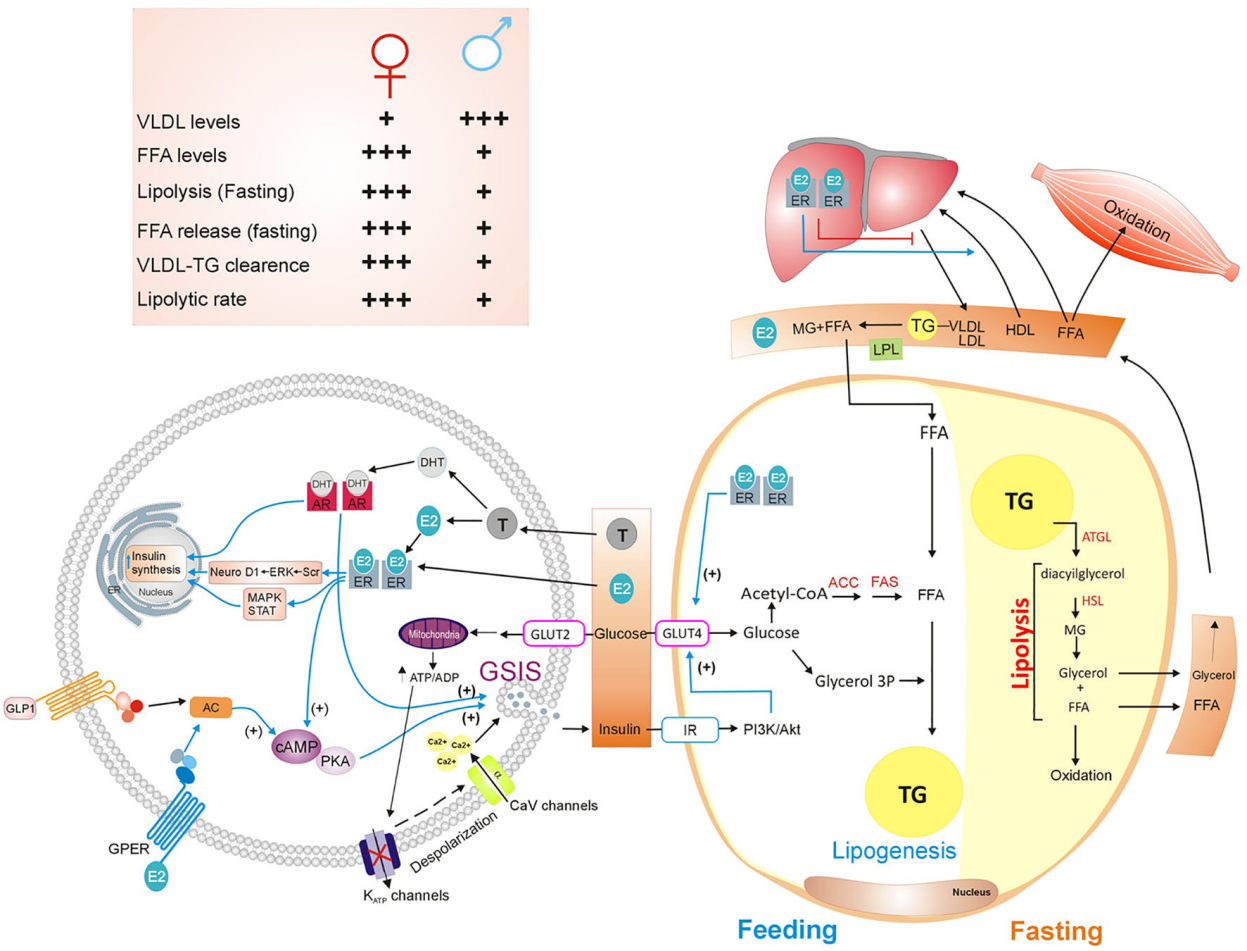
Sex hormones’ actions on lipids metabolism and insulin secretion. Lipid metabolism is a complex process that involves organs such as white adipose tissue, liver, pancreas, and muscle. After feeding, adipocytes convert carbohydrates into fatty acids and uptake plasma FFA released by the liver to store them as TG, in a process named lipogenesis. In contrast, during fasting, activation of lipolysis within adipocytes, breaks down TG into free fatty acids and glycerol, that are subsequently released from adipocyte. Muscle and liver oxidize FFA and glycerol. Insulin plays an important role by stimulating lipogenesis and inhibiting lipolysis. Moreover, sex hormones regulate insulin synthesis and GSIS (40–43). See attached list of abbreviations. Created with Corel Draw.

Adipose tissue regulates fat storage in triacylglycerols (TG) and releases FFA in an insulin-dependent manner. After a meal, adipocytes secrete lipoprotein lipase (LPL), which inserted in the plasma membrane of endothelial cells. LPL hydrolyses TG from chylomicrons and very low-density lipoproteins (VLDL) producing FFA and monoacylglycerol. Both lipids are internalized and re-esterified into TG by adipocytes. It is well documented that VLDL levels are higher in men than in women. On the other hand, women have higher VLDL–TG clearance rate than men (40) (**Figure 2**).

Lipolysis requires lipases enzymes such as ATGL, HSL, and MGL. Esterification and oxidation of lipids vary in fasting and exercise conditions. During fasting, lipolysis and FFA release are significantly greater in women than men (44). In the postprandial state, fatty acid oxidation is similar between women and men; however, when exercising, fatty acid oxidation is higher in women than men (40) (**Figure 2**).

Interestingly, despite having an increased lipogenic rate, the average size of female adipocytes is smaller than that of male adipocytes. Several studies have shown that female adipocytes have an increased lipolytic rate than male adipocytes (45). As a result, women have higher FFA serum levels than men. However, there are no significant differences in FFA serum levels between male and female mice. This observation supports the notion of a higher metabolic turnover of lipids in female visceral adipocytes, leading to a decreased fat accumulation in visceral depots compared to males (39).

Men and women have different adipokines secretion, mainly leptin and adiponectin. Plasma levels of both adipokines are higher in women than in men (46); they associate with the adipose tissue distribution and adipocyte size. White adipose tissue (WAT) is the main secretor of leptin. This hormone is a powerful catabolic signal in the brain that reduces food intake and increases energy expenditure (47). Interestingly, there is an association between high estrogen levels and increased leptin sensitivity in the brain (48).

Sex differences in adipose tissue distribution could be due, at least in part, to the effects of sexual hormones. In addition to the role of sex hormones on metabolism, recent reports point out that genes located on the X chromosome are associated with adiposity control (49). The FCG (four-core genotype) model generates mice with four combinations of gonads and sex chromosomes: XX female, XX male, XY female, and XY male mice, through the translocation of the *Sry* gene. Experimental evidence obtained from this model revealed a sex-chromosome complement contribution (50). Mice with two X chromosomes have an increased body fat proportion, independently of the gonadal steroid hormones.

Moreover, gonadectomized adult mice with XX chromosomes showed an augmented food intake, a rapid weight gain, and a higher body fat content on HFD than XY mice (51).

The mechanisms that underlie the effects of sex chromosomes on lipid metabolism and obesity are unknown. However, the sex-chromosome complement, overall and tissue-specific miRNA levels, the X chromosome imprinting, and the X inactivation escapee genes may be involved in the modulation of autosomal gene expression (51–54).

### Estrogens Actions on Lipid Metabolism

Sex hormones play a role in the regulation of adipose tissue function and whole-body insulin sensitivity. Estrogens have an essential role in modulating lipid metabolism through different estrogen receptors (ERα, ERβ, and GPER, also called Gpr30) expressed in the liver, adipose tissue, gut, and the central nervous system. Estrogens regulate the movement of FFA to adipose tissue from lipoproteins (chylomicrons and VLDL rich in TG) from the liver and the gut (41) (**Figure 2**).

Estrogen signaling may improve nutrient storage in the subcutaneous fat in women by increasing insulin and adiponectin sensitivity and promoting pre-adipocyte differentiation to white adipocytes. The relative redistribution in body fat from subcutaneous to visceral depots associates with decreased estrogens during menopause. Furthermore, castration of male mice enhances insulin sensitivity and increases adipocyte lipolytic rate (40, 41).

The ERα expression in female adipose tissue is higher than in males, and it correlates with increased insulin sensitivity in females compared to males. Systemic and adipose tissue-specific *Esr1* knockout mice develop insulin resistance (55).

Hepatic estrogen signaling in humans might also contribute to sex differences in cholesterol metabolism because estrogens promote hepatic reverse cholesterol transport, which is the process of cholesterol removal from peripheral tissues. This process culminates with cholesterol delivery to the liver. In addition, estradiol promotes the hepatic conversion of cholesterol into bile acids, and secretion into the bile duct (56) (**Figure 2**).

In macrophages, estradiol-esters enhance cholesterol efflux capacity in high-density lipoprotein (HDL), when estrogen levels rise during the proestrus phase (57).

The exact contribution of each estrogen receptor related to lipid metabolism is not well defined in humans. However, transcriptional activation of ERα by 17β-estradiol binding could regulate over 1000 genes containing estrogen response elements (EREs) and are mainly related to lipid metabolism pathways. Estrogen regulation of lipid-related pathways in mice may vary depending on the estrous cycle phase (58, 59).

Moreover, estrogens may regulate hepatic lipid metabolism by serine palmitoylation of caveolin-1 and association with membrane-associated ERα and ERβ, which activate the ERK1/2 and PI3K pathways. Furthermore, estrogens activate GPER, which increases intracellular cAMP and Ca^2+^, and is related to low-density lipoprotein (LDL) metabolism in mice (41).

### The Role of Estrogens on Insulin Secretion and Insulin Sensitivity

Insulin is a critical regulator of energy metabolism. Current evidence shows sex-related differences in beta-cell insulin secretion and the insulin signaling pathway in other tissues (60–64).

Studies in humans, using the hyperinsulinemic-euglycemic clamp technique, indicate that women are more sensitive to insulin, although they have a lower tolerance to glucose than men (65, 66). In addition, testosterone deficiency predisposes men to visceral obesity and impairs insulin sensitivity (67, 68). Moreover, testosterone excess predisposes women to obesity and hyperglycemia (68). Furthermore PCOS, the leading cause of androgen excess, predisposes women to develop T2DM (69).

In healthy men and hyperandrogenic women, testosterone is the most abundant androgen (70), while 17β-estradiol or E2 is the most abundant estrogen in healthy premenopausal women (71). Moreover, testosterone undergoes conversion to either dihydrotestosterone (DHT) or E2 in target tissues, *via* 5α-reductase and aromatase enzymes, respectively. Interestingly, a recent study showed for the first time that pancreatic beta cells could produce androgens and estrogens from circulating testosterone (70).

These sexual hormones can enter the blood stream and interact with their receptors. Androgens receptors (AR) are present in female and male pancreatic beta-cells (42), and three estrogens receptors (ERα, ERβ and GPER) had been identified in rodent and human beta-cells (72). In classic target tissues of sexual hormones, these receptors act as ligand-activated transcriptional factors. In pancreatic beta-cells, ERs and ARs reside mainly in extranuclear locations (71) (**Figure 2**).

Accumulated evidence suggests that estrogens (17β-estradiol) and androgens (testosterone) modulate insulin secretion in a sexually dimorphic manner (42, 71). Previous work in our laboratory with pancreatic islets of female rats observed higher insulin secretion rates when comparted with those of male rats (73). In addition, variations in pancreatic insulin mRNA levels and serum insulin levels have been observed during the estrous cycle, suggesting that sex steroid hormones could modulate insulin secretion (74, 75).

In addition, testosterone deficiency predisposes to pancreatic beta-cell dysfunction and insulin deficiency (42). In contrast, DHT enhances glucose-stimulated insulin secretion (GSIS) in cultured male islets, and this effect is abolished in male mice lacking AR in beta-cells (βARKO) (76).

On the other hand, female rats exposed to DHT show hyperinsulinemia due to increased insulin gene transcription in pancreatic beta cells (77). While, estrogens promote insulin-producing beta-cell function by increasing their electrical activity, survival, and proliferation (43).

Testosterone may increase GSIS in pancreatic beta-cells *via* AR and GLP-1 receptors by increasing intracellular cAMP levels and amplifying the incretin effects of GLP-1 (76). GSIS in pancreatic beta-cells begins with glucose internalization and metabolism, which increases ATP levels and membrane depolarization that triggers [Ca2+] influx (78). Interestingly, testosterone action on GSIS is independent of increases in intracellular ATP levels (76) (**Figure 2**).

In contrast, ERs exert their effects *via* cytosolic interactions with kinases such as Src and ERK that subsequently could activate Neuro D1 an insulinotropic transcription factor. Moreover, ERs may activate AMPK or through transcription factors such as the STAT family to induce some of their effects (79). GPER activation protects pancreatic beta cells from lipid accumulation and promotes their survival (71). Furthermore, activation of GPER also increases the GSIS *via* activation of ERK signaling pathway (80) (**Figure 2**).

## Sexual Dimorphism in Adipose Tissue Dysfunction

Sex differences in the molecular mechanisms that control adipose tissue´s function and its relationship with other organs have clinical implications. They participate in the development of obesity, dyslipidemia, insulin resistance, and hypertension favoring the establishment of metabolic syndrome and increasing the risk of developing T2DM and CVDs (2).

In 2016, the World Health Organization (WHO) reported that around 13% of the world’s adult population (11% of men and 15% of women) were obese. Furthermore, even though women have significantly more body fat accumulation, the prevalence of diabetes and early glucose metabolism abnormalities is higher in men than in women. The last global estimates published by the International Diabetes Federation showed sexual differences in worldwide diabetes prevalence in the adult population, 9.1% in men compared to 8.4% in women (1). Epidemiologic studies show that diabetes and pre-diabetes are less prevalent in pre- and peri-menopausal women than age-adjusted men (2).

Furthermore, it has been well established that after menopause, the decline in estrogen levels produces an increase of visceral fat, associated with insulin resistance and an increased cardiovascular risk (41). Several studies had observed that premenopausal women have lower incidence and severity of hypertension. Thus they have a lower incidence of myocardial infarction and CVDs than men (81).

In consequence, response to treatment, complications of metabolic diseases, and mortality have shown sex-specific differences.

### Obesity, Dyslipidemia, and Inflammation

Obesity is defined as an excessive accumulation of fat that can be harmful to health. According to WHO, a person with a BMI ≥ 30 is considered obese. The most common cause is a positive balance between caloric intake and energy expenditure. In obesity, there is an excessive accumulation of lipids in WAT, which causes a rise in adipocyte number (hyperplasia) or an enhanced lipid accumulation (hypertrophy), generating several metabolic alterations (82, 83).

The higher visceral adiposity observed in men is associated with elevated postprandial insulin, FFA, and TG levels (84). Visceral adipocytes are more sensitive to catecholamine-induced lipolysis and less susceptible to insulin´s antilipolytic effect than subcutaneous adipocytes. This leads to an increased FFA delivery to the portal system, resulting in increased gluconeogenesis, VLDL secretion, and decreased hepatic insulin clearance. This way, higher lipolytic activity in visceral fat and its direct connection with the liver is associated with increased dyslipidemia and insulin resistance (85).

Once adipocyte storage and mitochondrial oxidative capacity are overwhelmed, lipids in excess accumulate in non-adipose tissues such as the liver, muscle, and pancreas (86), creating a condition that is known as lipotoxicity.

In obesity, men show a higher VLDL production than women do; in part, secondary to a lower FFA delivery to the liver due to enhanced FFA clearance by muscle in women. Accordingly, male HFD-fed mice have increased serum TG levels relative to females (40, 63). Intramuscular TG accumulation is also associated with insulin resistance and impaired glucose disposal, and it is only observed in men (87).

Morbid obesity usually causes an aberrant accumulation of TG in the liver, which leads to hepatic steatosis and further impairs systemic fat metabolism (64, 88).

During obesity, adipocytes constantly die by necrosis, caused by physical stress, hypoxia, mitochondrial dysfunction, and by the production of reactive oxygen species (ROS) due to excessive levels of FFA. After necrosis, the adipocyte debris are recognized by monocytes and antigen-presenting cells (APC), like macrophages and dendritic cells, present in the adipose tissue. Once activated, these cells start a low-grade inflammation, a process known as meta-inflammation (89). Accordingly, there is a significant increase in WAT macrophage accumulation in male HFD-fed mouse, compared to females (63).

This meta-inflammation observed in obesity differs between sexes at the molecular, cellular, and systemic level. At a molecular level, adipokines and cytokines secreted by adipose tissue respond to inflammation and act as endocrine molecules, usually through JAK/STAT, NF-κB, and JNK kinase signaling pathways. These pathways are essential to regulate body homeostasis, but once deregulated, they contribute to the differential development of insulin resistance between males and females (90).

There is a predominantly anti-inflammatory profile in healthy adipose tissue, the most secreted cytokines are interleukin-4, -13, and -10 (IL-4, IL-13, and IL-10). On the other hand, the resident immune cells in WAT switch to a pro-inflammatory profile and release cytokines like TNFα, IL-1β, and IL-6. Regarding to the sexual dimorphic cytokine production in obese humans, in men, peripheral mononuclear cells produce more TNFα and less IL-10 than women (91). In HFD-induced obesity murine models, peritoneal macrophages from male mice express higher TLR4 levels and CXCL 10 compared to female mice. This fact means more macrophages M1 activation and more immune cells attraction to inflammation site and, constitutive production of TNFα (92).

In female C57/BL6 mice, 17β-estradiol stimulates an anti-inflammatory response directly in adipocytes by reducing the production of TNFα and subsequent activation of NF-κB (93).

According to the differences previously mentioned between men and women, the obesity-related meta-inflammation and treatment of obesity complications are more complex than previously thought.

### Sexual Dimorphism in Insulin Resistance

Insulin resistance is a condition in which there is a reduced response of organs to insulin actions, manifested by mild hyperglycemia (less than 125 mg/dL) and hyperinsulinemia in fasting and postprandial states. In murine models of insulin resistance, there is an increase in the production and release of hepatic glucose and a glycogen synthesis decrease in muscle, which increases blood glucose in fasting (94). Plasma insulin levels are elevated during fasting due to elevated glucose beta-cell stimulation and or in response to meta-inflammation because even with average glucose values, there is hyperinsulinemia (95). There are alterations in lipid metabolism in adipose tissue and liver in rats with insulin resistance (64, 95). These alterations reflect an increase in FFA levels in circulation due to the loss of lipolysis inhibition in adipose tissue in these animals (88).

Release of FFA and cytokines from WAT induces accumulation of ectopic fat in muscle and the liver, as well as inflammation and insulin signaling defects in other tissues (6, 64).

In states such as early development, adolescence, or pregnancy, and even in some infectious processes, insulin resistance is physiological because it is an adaptive response to high energy demand (7, 96). However, there is sufficient evidence that obesity-related insulin resistance is an early and determining risk factor in establishing metabolic syndrome, and consequently, T2DM, and some types of cancer (2, 6, 64, 94).

Insulin resistance is sexually dimorphic. In mice models of HFD-induced obesity, despite similar levels of obesity in both sexes, males display higher fasting plasma glucose levels and develop more severe glucose intolerance and insulin resistance than females (63). Male rodents fed with HFD develop insulin resistance within three weeks, but females are less prone to the metabolic disturbances caused by HFD, suggesting that females are less susceptible to fatty acids-induced systemic insulin resistance (65).

Intralipid infusion has been used as a model to investigate lipid-induced insulin resistance in rodents and humans. In two hours, intralipid infusion reduced the phosphorylation of IRS1, PI3K activity, and the insulin-stimulated glucose uptake in male but not in female rats. In healthy individuals, intralipid infusion causes less insulin resistance in women than in men. This insulin resistance does not impair insulin or AMPK signaling in muscle and subcutaneous fat. It does not cause accumulation of lipids in muscle, inflammation, or direct inhibition of GLUT4 activity in women. Instead, it turns a higher lactate release and lower glucose oxidation that may suggest a “metabolic switch” of glucose metabolism to lipid metabolism induced by intralipid infusion, particularly in women (97).

Obesity is associated with an enhanced oxidative function and increased cellular oxidative stress. Mitochondrial dysfunction in WAT derived from fatty acid accumulation leads to a dysregulation of adipokine secretion, affecting insulin sensitivity. Recent investigations suggest that ER elicits estrogen´s metabolic effects by mitochondrial mechanisms involved in the regulation of insulin signaling (98). A crucial link between mitochondrial dysfunction and insulin sensitivity is adiponectin production. Adiponectin plays a role as an insulin-sensitizing factor. HFD induces mitochondrial differentiation in females and a greater retroperitoneal WAT expandability and decreased adiponectin levels. Surprisingly, in females, HFD-induced changes allow better systemic insulin sensitivity and delay lipotoxicity development than male rats. This sexual dimorphism suggests different physiological strategies between males and females to maintain energetic and metabolic homeostasis in response to the high uptake of lipids (99).

In estrogen-poor conditions such as menopause, ovariectomy, and aromatase deficiency, insulin sensitivity is decreased (100). HFD-fed ovariectomized female mice exhibit a reduced insulin sensitivity due to an increased TNFα synthesis. TNFα activates phosphatases such as PTP1B, which can dephosphorylate IRS2 and downregulate Akt, interfering with GLUT4 translocation to the plasma membrane (101). Conversely, estradiol supplementation reduces inflammation, improves insulin sensitivity, and down-regulates TNFα and IL-6 expression (102).

#### Sex-Related Differences in the Insulin Signaling Pathway

**Figure 3:**
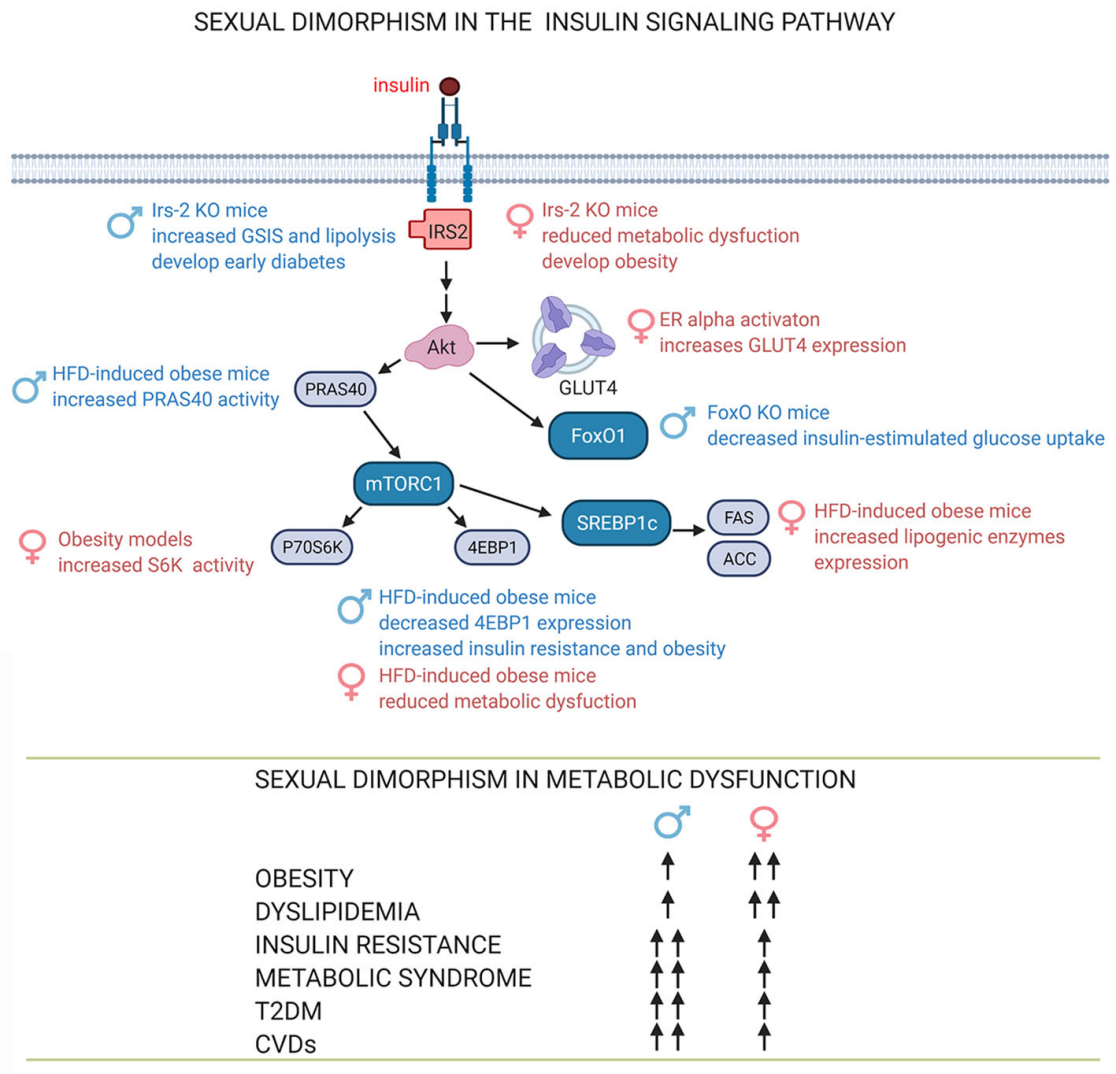
Sexual dimorphism in the insulin signaling pathway. The sex-dependent function of proteins in the insulin signaling pathway involves sex-dependent differences in adipose tissue function and consequently in the development and progression of obesity, dyslipidemia and insulin resistance, signs associated with metabolic diseases such as metabolic syndrome, T2DM, CVDs (39, 60, 61, 63, 64, 103). See attached list of abbreviations. Created with BioRender.com.

The sexual dimorphism in adipose tissue’s the physiology and pathophysiology has been extensively studied. However, there is not much data related to sex-related differences in the molecular mechanisms of insulin actions and the consequences of its dysregulation. Here we present some of the findings reported so far (**Figure 3**).

#### IRS2

IRS2 is known to have an essential role in hypothalamic regulation of appetite and obesity (104). *Irs*-2 deletion in mice causes diabetes and has a sexually dimorphic phenotype. Males *Irs-2^-/-^* mice develop diabetes at 12 weeks of age, while in females, this deletion generates obesity and a slower progression of this disease. García-Barrado and colls. (2011) explored beta-cell function and lipolysis as a possible cause of sex-related differences in this model. They reported an increased GSIS on islets from male *Irs-2^-/-^* mice compared to wildtype controls due to lower expression of α_2_-AR and attenuation of their inhibitory role on insulin secretion, which favors beta-cells damage and their subsequent dysfunction in males.

On the other hand, adipocytes from both male and female *Irs-2^-/-^* mice show resistance to the anti-lipolytic effects of insulin. However, female *Irs-2^-/-^* mice also present resistance to catecholamines, impairment of cAMP synthesis, and in consequence, downregulation of PKA, which drives lipolysis. The HSL lipolytic enzyme activity is blunted in adipose tissue of female *Irs-2 ^-/-^* mice and this decreases the adverse effects of circulating lipids in females. These results suggest that IRS2 may play a sexually dimorphic role in the regulating insulin sensitivity in adipose tissue function (103).

#### mTORC1 Signaling Pathway

The activation of mTORC1 through Akt promotes the phosphorylation of S6K and 4E-BP which induces ribosomal biogenesis and mRNA translation, respectively. The dysregulation of mTORC1 signaling in tissues of obese individuals and murine models is related to insulin resistance (64, 105). Moreover, there is an increase of S6K1 signaling in tissues from diabetic individuals, and there is evidence that S6K1 removal in mice protects them from diet-induced obesity and insulin resistance (106).

Tsai and collaborators (2016) have identified gender-specific differences in the mTORC1 signaling pathway in mice fed with HFD and obesity associated with aging. The mRNA expression and protein level of 4E-BP1 decreases under these conditions in the liver, skeletal muscle, and adipose tissue of males, but not in females. A transgenic mouse model that over-expresses 4E-BP1 demonstrated that this protein protects male mice against obesity and HFD-induced insulin resistance. As a result, 4E-BP1 is a gender-specific obesity suppressor that regulates insulin sensitivity (63).

Among other mTORC1 targets examined, in aging female tissues, there is an upregulation of S6K1 activity. The phosphorylation of PRAS40 by mTORC1 on Ser183 increases visceral fat of male HFD-treatment mice compared to female mice (63).

Finally, high-sucrose diet induces metabolic syndrome in *Wistar* rats, and insulin resistance associated with sex-dependent differences in the Akt-mTORC1-S6K signaling axis (64).

#### GLUT4

The insulin-sensitive glucose transporter GLUT4 is translocated to the plasma membrane in response to insulin stimulation in adipose tissue and skeletal muscle. Recent studies indicate that this is also true in the hippocampus (107, 108).

Adipocytes from visceral and subcutaneous adipose tissue from female mice had higher mRNA and protein levels of GLUT4. In addition, they also display increased amounts of key lipogenic enzymes such as FAS and ACC than male adipocytes (39).

CEBPA and PPARγ are the main transcriptional factors that regulate adipocyte differentiation. These transcriptional factors regulate the expression of the *Slc2a4* gene, which codes for GLUT4. Interestingly, although PPARγ upregulates genes related to adipocyte differentiation, it downregulates *Slc2a4* expression. *Cebpa* expression increases during adipocyte differentiation. Estradiol increases the expression of *Cepba* mRNA, CEBPA nuclear content, and its binding to the *Slc2a4* promoter (61). This way, estradiol stimulates the differentiation of 3T3-L1 adipocytes. ERα activation in adipose tissue increases *Slc2a4* expression, GLUT4 translocation to the plasma membrane, and subsequent glucose uptake (109) through the ERα/CEBPA-mediated pathway, thus revealing a mechanism by which estradiol can modulate adipogenesis and GLUT4 expression (61) (**Figure 2**).

#### FoxO

FoxOs are transcription factors ubiquitously expressed. They control cellular differentiation, muscle growth, metabolism, and tumor suppression pathways. FoxOs are directly inhibited by the actions of insulin in its target tissues (6).

There is a decreased insulin-stimulated Akt activation in males in a model of mice of muscle-specific FoxO1/3/4 triple knockout (TKO); Akt2 mRNA and protein levels are reduced, as well as were protein and phosphorylation levels of insulin receptor and IRS2 mRNA. These changes contributed to a decreased insulin-stimulated glucose uptake in the muscle of male TKO mice, altering glucose homeostasis. In contrast, female TKO mice maintained normal Akt2 levels, unchanged levels of insulin-mediated Akt phosphorylation, and normal glucose uptake in muscle compared to those of controls. Thus, FoxO deletion in skeletal muscle reveals sex-dependent differences in Akt2 associated with impaired insulin signaling in male mice muscle, but not in females. Penniman and collaborators suggest that FoxO promotes insulin sensitivity in male mice muscle, probably due to an increased Akt2 expression (60).

## Conclusion

There is ample evidence from animal models and humans that males and females are different phenotypically and metabolically at cellular and molecular level. Females are protected against obesity–induced insulin resistance due to sex hormones and sex-specific gene expression in adipose tissue. Furthermore, there are variations between males and females in insulin actions through of the insulin signaling pathway. Although sex is a critical factor in the prevalence and severity of metabolic diseases, males have historically been used in scientific research scientific to avoid female sex hormones actions along the estral cycle. However, sex should be considered when investigating molecular processes related to insulin signaling and related metabolic pathways in healthy metabolism and disease (**Figure 3**). This new consideration could lead to more efficient and urgently needed sex-targeted therapies to treat obesity and its comorbidities.

## Author Contributions

RO-H wrote and edited the initial draft of the manuscript. MV, CL, RE, and MH wrote, edited, and critically reviewed the manuscript. All authors contributed to the article and approved the submitted version.

## Funding

This work was supported by DGAPA-PAPIIT (IN208720) and CONACYT (FORDECYT-PRONACES/568492/2020) to MH, and CONACYT (240108).

## Conflict of Interest

The authors declare that the research was conducted in the absence of any commercial or financial relationships that could be construed as a potential conflict of interest.

## Glossary

**Table:**

4E-BP	Eukaryotic translation initiation factor 4E binding protein
5´AMP	Adenosine 5´-monophosphate
AC	Adenilate cyclase
ACC	Acetyl-CoA carboxylase
Akt	Protein kinase B
AMPK	AMP-activated protein kinase
AR	Androgen receptors
ATGL	Adipose triglyceride lipase
ATP	Adenosine triphosphate
cAMP	Cyclic adenosine monophosphate
CEBPA	Estrogen receptor 1/CCAAT/enhancer-binding protein alpha
CXCL 10	CXC-chemokine ligand 10
DHT	Dihydrotestosterone
E2	17β-estradiol
ERK	Extracellular signal-regulated kinase
ERα	Estrogen receptor α
ERβ	Estrogen receptor β
*Esr1*	Estrogen receptor 1 gene
FAS	Fatty acid synthase
FoxO1	Forkhead box protein O1
FSH	Follicle stimulating hormone
G6Pase	Glucose 6-phosphatase
GDP	Guanosine diphosphate
GLP-1	Glucagon-like peptide-1
GLUT2	Glucotransporter 2
GLUT4	Glucotransporter 4
GPER	G-protein coupled estrogen receptor
Grb2	Growth factor receptor-bound protein 2
GSK3β	Glycogen synthase kinase 3β
GTP	Guanosine 5´triphosphate
HSL	Hormone-sensitive lipase
*Igfr*	Insulin-like growth factor receptor gene
*Insr*	Insulin receptor gene
IR	Insulin receptor
IRS1	Insulin receptor substrate 1
IRS2	Insulin receptor substrate 2
*Irs-2*	Insulin receptor substrate 2 gene
JAK	Janus kinase
JNK	c-Jun N-terminal kinase
LH	Luteinizing hormone
MAPK	Mitogen-Activated Protein Kinase
MEK	MAPK ERK kinase
MGL	Monoacylglycerol lipase
miRNA	Micro RNA
mRNA	Messenger RNA
mTOR	mammalian Target Of Rapamycin
mTORC1	mammalian Target Of Rapamycin Complex 1
mTORC2	mammalian Target Of Rapamycin Complex 2
NF-κB	Nuclear factor-κB
PDE3B	Phosphodiesterase 3B
PDK1	Phosphoinositide-dependent kinase-1
PEPCK	Phosphoenolpyruvate carboxykinase
PGC1α	Peroxisome proliferator-activated receptor-gamma coactivator-1α
PI(3,4,5)P3	Phosphatidylinositol 3,4,5-trisphosphate
PI(4,5)P2	Phosphatidylinositol 4,5-bisphosphate
PI3K	Phosphatidylinositol–3 kinase
PKA	cAMP-dependent protein kinase
PPARγ	Peroxisome proliferator-activated receptor-gamma
PRAS40	Proline-rich Akt substrate
PTP1B	Protein tyrosine phosphatase 1B
Raf-1	RAF proto-oncogene serine/threonine-protein kinase
Raptor	Regulatory-associated protein of mTOR
Ras	Ras proteins (small guanosine triphosphatases)
Rheb	Ras homolog enriched in brain
S6K	Ribosomal S6 kinase
ShC	Src homology and collagen protein
*Slc2a4*	Solute carrier family 2-member 4 gene
SOS	Guanin exchange factor son of sevenless
Src	Proto-oncogene tyrosine-protein kinase
SREBP1c	Sterol regulatory element-binding protein 1c
*Sry*	Sex determining region Y gene
STAT	Signal transducer and activator of transcription
TBC1D4	TBC1 domain family member 4
T	Testosterone
TG	Triacylglycerols
TLR4	Toll-like receptor 4
TNFα	Tumor necrosis factor α
TSC1/2	Tuberous sclerosis protein ½
α_2_-AR	α_2_-adrenoceptors

## References

[1] Tramunt B Smati S Grandgeorge N Lenfant F Arnal J-F Montagner A. Sex Differences in Metabolic Regulation and Diabetes Susceptibility. Diabetologia (2020) 63(3):453–61. 10.1007/s00125-019-05040-3 PMC6997275 31754750

[2] Kautzky-Willer A Harreiter J Pacini G. Sex and Gender Differences in Risk, Pathophysiology and Complications of Type 2 Diabetes Mellitus. Endocr Rev (2016) 37(3):278–316. 10.1210/er.2015-1137 27159875 PMC4890267

[3] Scheja L Heeren J. The Endocrine Function of Adipose Tissues in Health and Cardiometabolic Disease. Nat Rev Endocrinol (2019) 15(9):507–24. 10.1038/s41574-019-0230-6 31296970

[4] Smith U Kahn BB. Adipose Tissue Regulates Insulin Sensitivity: Role of Adipogenesis, *De Novo* Lipogenesis and Novel Lipids. J Intern Med (2016) 280(5):465–75. 10.1111/joim.12540 PMC5218584 27699898

[5] Goossens GH Jocken JWE Blaak EE. Sexual Dimorphism in Cardiometabolic Health: The Role of Adipose Tissue, Muscle and Liver. Nat Rev Endocrinol (2021) 17(1):47–66. 10.1038/s41574-020-00431-8 33173188

[6] Petersen MC Shulman GI. Mechanisms of Insulin Action and Insulin Resistance. Physiol Rev (2018) 98(4):2133–223. 10.1152/physrev.00063.2017 PMC6170977 30067154

[7] Haeusler RA McGraw TE Accili D. Biochemical and Cellular Properties of Insulin Receptor Signalling. Nat Rev Mol Cell Biol (2018) 19(1):31–44. 10.1038/nrm.2017.89 28974775 PMC5894887

[8] Hiriart M Velasco M Larqué C Diaz-Garcia CM. Metabolic Syndrome and Ionic Channels in Pancreatic Beta Cells. Vitam Horm (2014) 95:87–114. 10.1016/B978-0-12-800174-5.00004-1 24559915

[9] Boucher J Kleinridders A Kahn CR. Insulin Receptor Signaling in Normal and Insulin-Resistant States. Cold Spring Harb Perspect Biol (2014) 6(1):a009191. 10.1101/cshperspect.a009191 24384568 PMC3941218

[10] Maggi A Della Torre S. Sex, Metabolism and Health. Mol Metab (2018) 15:3–7. 10.1016/j.molmet.2018.02.012 29550349 PMC6066735

[11] Peippo J Bredbacka P. Sex-Related Growth Rate Differences in Mouse Preimplantation Embryos *in Vivo* and In Vitro. Mol Reprod Dev (1995) 40(1):56–61. 10.1002/mrd.1080400108 7702870

[12] Ray PF Conaghan J Winston RM Handyside AH. Increased Number of Cells and Metabolic Activity in Male Human Preimplantation Embryos Following *in Vitro* Fertilization. J Reprod Fertil (1995) 104(1):165–71. 10.1530/jrf.0.1040165 7636798

[13] Nef S Verma-Kurvari S Merenmies J Vassalli J-D Efstratiadis A Accili D. Testis Determination Requires Insulin Receptor Family Function in Mice. Nature (2003) 426(6964):291–5. 10.1038/nature02059 14628051

[14] Neirijnck Y Calvel P Kilcoyne KR Kühne F Stévant I Griffeth RJ. Insulin and IGF1 Receptors Are Essential for the Development and Steroidogenic Function of Adult Leydig Cells. FASEB J (2018) 32(6):3321–35. 10.1096/fj.201700769RR 29401624

[15] Pitetti J-L Calvel P Zimmermann C Conne B Papaioannou MD Aubry F. An Essential Role for Insulin and IGF1 Receptors in Regulating Sertoli Cell Proliferation, Testis Size, and FSH Action in Mice. Mol Endocrinol (2013) 27(5):814–27. 10.1210/me.2012-1258 PMC5416760 23518924

[16] Griffeth RJ Carretero J Burks DJ. Insulin Receptor Substrate 2 Is Required for Testicular Development. PloS One (2013) 8(5):e62103. 10.1371/journal.pone.0062103 23741292 PMC3669358

[17] Kim E Seok HH Lee S-Y Lee DR Moon J Yoon TK. Correlation Between Expression of Glucose Transporters in Granulosa Cells and Oocyte Quality in Women With Polycystic Ovary Syndrome. Endocrinol Metab (Seoul) (2014) 29(1):40–7. 10.3803/EnM.2014.29.1.40 PMC3970285 24741453

[18] Rabiee AR Lean IJ Gooden JM Miller BG Scaramuzzi RJ. An Evaluation of Transovarian Uptake of Metabolites Using Arterio-Venous Difference Methods in Dairy Cattle. Anim Reprod Sci (1997) 48(1):9–25. 10.1016/S0378-4320(97)00032-8 9412729

[19] Escott GM Jacobus AP Loss ES. Pi3k-Dependent Actions of Insulin and IGF-I on Seminiferous Tubules From Immature Rats. Pflugers Arch (2013) 465(10):1497–505. 10.1007/s00424-013-1287-z 23636775

[20] Sugiura K Eppig JJ. Society for Reproductive Biology Founders’ Lecture 2005. Control of Metabolic Cooperativity Between Oocytes and Their Companion Granulosa Cells by Mouse Oocytes. Reprod Fertil Dev (2005) 17(7):667–74. 10.1071/rd05071 16364219

[21] van der Steeg JW Steures P Eijkemans MJC Habbema JDF Hompes PGA Burggraaff JM. Obesity Affects Spontaneous Pregnancy Chances in Subfertile, Ovulatory Women. Hum Reprod (2008) 23(2):324–8. 10.1093/humrep/dem371 18077317

[22] Dunaif A. Insulin Resistance and the Polycystic Ovary Syndrome: Mechanism and Implications for Pathogenesis. Endocr Rev (1997) 18(6):774–800. 10.1210/edrv.18.6.0318 9408743

[23] Grieger JA Grzeskowiak LE Smithers LG Bianco-Miotto T Leemaqz SY Andraweera P. Metabolic Syndrome and Time to Pregnancy: A Retrospective Study of Nulliparous Women. BJOG (2019) 126(7):852–62. 10.1210/edrv.18.6.0318 30734474

[24] Leisegang K Bouic PJD Menkveld R Henkel RR. Obesity Is Associated With Increased Seminal Insulin and Leptin Alongside Reduced Fertility Parameters in a Controlled Male Cohort. Reprod Biol Endocrinol (2014) 12:34. 10.1186/1477-7827-12-34 24885899 PMC4019561

[25] Samoto T Maruo T Ladines-Llave CA Matsuo H Deguchi J Barnea ER. Insulin Receptor Expression in Follicular and Stromal Compartments of the Human Ovary Over the Course of Follicular Growth, Regression and Atresia. Endocr J (1993) 40(6):715–26. 10.1507/endocrj.40.715 7951542

[26] Bhatia B Price CA. Insulin Alters the Effects of Follicle Stimulating Hormone on Aromatase in Bovine Granulosa Cells In Vitro. Steroids (2001) 66(6):511–9. 10.1016/s0039-128x(00)00218-x 11182140

[27] Liu Z Rudd MD Hernandez-Gonzalez I Gonzalez-Robayna I Fan H-Y Zeleznik AJ. FSH and FOXO1 Regulate Genes in the Sterol/Steroid and Lipid Biosynthetic Pathways in Granulosa Cells. Mol Endocrinol (2009) 23(5):649–61. 10.1210/me.2008-0412 PMC2675958 19196834

[28] Bergh C Carlsson B Olsson JH Selleskog U Hillensjö T. Regulation of Androgen Production in Cultured Human Thecal Cells by Insulin-Like Growth Factor I and Insulin. Fertil Steril (1993) 59(2):323–31. 10.1016/s0015-0282(16)55675-1 8425626

[29] Palaniappan M Menon B Menon KMJ. Stimulatory Effect of Insulin on Theca-Interstitial Cell Proliferation and Cell Cycle Regulatory Proteins Through Mtorc1 Dependent Pathway. Mol Cell Endocrinol (2013) 366(1):81–9. 10.1016/j.mce.2012.12.004 PMC3552006 23261705

[30] Ratchford AM Chang AS Chi MM-Y Sheridan R Moley KH. Maternal Diabetes Adversely Affects AMP-Activated Protein Kinase Activity and Cellular Metabolism in Murine Oocytes. Am J Physiol Endocrinol Metab (2007) 293(5):E1198–206. 10.1152/ajpendo.00097.2007 17684106

[31] Wang Q Ratchford AM Chi MM-Y Schoeller E Frolova A Schedl T. Maternal Diabetes Causes Mitochondrial Dysfunction and Meiotic Defects in Murine Oocytes. Mol Endocrinol (2009) 23(10):1603–12. 10.1210/me.2009-0033 PMC2754898 19574447

[32] Ahn SW Gang G-T Kim YD Ahn R-S Harris RA Lee C-H. Insulin Directly Regulates Steroidogenesis *Via* Induction of the Orphan Nuclear Receptor DAX-1 in Testicular Leydig Cells. J Biol Chem (2013) 288(22):15937–46. 10.1074/jbc.M113.451773 PMC3668749 23589295

[33] Schianca GPC Fra GP Brustia F Bellan M Pirovano A Gualerzi A. Testosterone Plasma Concentration Is Associated With Insulin Resistance in Male Hypertensive Patients. Exp Clin Endocrinol Diabetes (2017) 125(3):171–5. 10.1055/s-0042-121492 28073130

[34] Wells JCK. Sexual Dimorphism of Body Composition. Best Pract Res Clin Endocrinol Metab (2007) 21(3):415–30. 10.1016/j.beem.2007.04.007 17875489

[35] Dearden L Bouret SG Ozanne SE. Sex and Gender Differences in Developmental Programming of Metabolism. Mol Metab (2018) 15:8–19. 10.1016/j.molmet.2018.04.007 29773464 PMC6066743

[36] Karastergiou K Smith SR Greenberg AS Fried SK. Sex Differences in Human Adipose Tissues - the Biology of Pear Shape. Biol Sex Differ (2012) 3(1):13. 10.1186/2042-6410-3-13 22651247 PMC3411490

[37] Fuente-Martín E Argente-Arizón P Ros P Argente J Chowen JA. Sex Differences in Adipose Tissue: It is Not Only a Question of Quantity and Distribution. Adipocyte (2013) 2(3):128–34. 10.4161/adip.24075 PMC3756100 23991358

[38] Snijder MB Dekker JM Visser M Bouter LM Stehouwer CDA Yudkin JS. Trunk Fat and Leg Fat Have Independent and Opposite Associations With Fasting and Postload Glucose Levels: The Hoorn Study. Diabetes Care (2004) 27(2):372–7. 10.2337/diacare.27.2.372 14747216

[39] Macotela Y Boucher J Tran TT Kahn CR. Sex and Depot Differences in Adipocyte Insulin Sensitivity and Glucose Metabolism. Diabetes (2009) 58(4):803–12. 10.2337/db08-1054 PMC2661589 19136652

[40] Santosa S Jensen MD. The Sexual Dimorphism of Lipid Kinetics in Humans. Front Endocrinol (Lausanne) (2015) 6:103. 10.3389/fendo.2015.00103 26191040 PMC4489151

[41] Palmisano BT Zhu L Eckel RH Stafford JM. Sex Differences in Lipid and Lipoprotein Metabolism. Mol Metab (2018) 115:45–55. 10.1016/j.molmet.2018.05.008 PMC6066747 29858147

[42] Xu W Morford J Mauvais-Jarvis F. Emerging Role of Testosterone in Pancreatic β-Cell Function and Insulin Secretion. J Endocrinol (2019) JOE-18-0573.R1. 10.1530/JOE-18-0573 PMC6602868 30601759

[43] Mauvais-Jarvis F. Role of Sex Steroids in β Cell Function, Growth, and Survival. Trends Endocrinol Metab (2016) 27(12):844–55. 10.1016/j.tem.2016.08.008 PMC5116277 27640750

[44] Hedrington MS Davis SN. Sexual Dimorphism in Glucose and Lipid Metabolism During Fasting, Hypoglycemia, and Exercise. Front Endocrinol (Lausanne) (2015) 6:61. 10.3389/fendo.2015.00061 25964778 PMC4410598

[45] Mittendorfer B Horowitz JF Klein S. Gender Differences in Lipid and Glucose Kinetics During Short-Term Fasting. Am J Physiol Endocrinol Metab (2001) 281(6):E1333–9. 10.1152/ajpendo.2001.281.6.E1333 11701450

[46] Cnop M Havel PJ Utzschneider KM Carr DB Sinha MK Boyko EJ. Relationship of Adiponectin to Body Fat Distribution, Insulin Sensitivity and Plasma Lipoproteins: Evidence for Independent Roles of Age and Sex. Diabetologia (2003) 46(4):459–69. 10.1007/s00125-003-1074-z 12687327

[47] Myers MG Leibel RL Seeley RJ Schwartz MW. Obesity and Leptin Resistance: Distinguishing Cause From Effect. Trends Endocrinol Metab (2010) 21(11):643–51. 10.1016/j.tem.2010.08.002 PMC2967652 20846876

[48] Clegg DJ Brown LM Woods SC Benoit SC. Gonadal Hormones Determine Sensitivity to Central Leptin and Insulin. Diabetes (2006) 55(4):978–87. 10.2337/diabetes.55.04.06.db05-1339 16567519

[49] Chen X McClusky R Chen J Beaven SW Tontonoz P Arnold AP. The Number of X Chromosomes Causes Sex Differences in Adiposity in Mice. PloS Genet (2012) 8(5):e1002709. 10.1371/journal.pgen.1002709 22589744 PMC3349739

[50] Burgoyne PS Arnold AP. A Primer on the Use of Mouse Models for Identifying Direct Sex Chromosome Effects That Cause Sex Differences in non-Gonadal Tissues. Biol Sex Differ (2016) 7:68. 10.1186/s13293-016-0115-5 27999654 PMC5154145

[51] Zore T Palafox M Reue K. Sex Differences in Obesity, Lipid Metabolism, and Inflammation-A Role for the Sex Chromosomes? Mol Metab (2018) 15:35–44. 10.1016/j.molmet.2018.04.003 29706320 PMC6066740

[52] Link JC Hasin-Brumshtein Y Cantor RM Chen X Arnold AP Lusis AJ. Diet, Gonadal Sex, and Sex Chromosome Complement Influence White Adipose Tissue miRNA Expression. BMC Genomics (2017) 18(1):89. 10.1186/s12864-017-3484-1 28095800 PMC5240420

[53] Gershoni M Pietrokovski S. The Landscape of Sex-Differential Transcriptome and its Consequent Selection in Human Adults. BMC Biol (2017) 15(1):7. 10.1186/s12915-017-0352-z 28173793 PMC5297171

[54] Berletch JB Ma W Yang F Shendure J Noble WS Disteche CM. Escape From X Inactivation Varies in Mouse Tissues. PloS Genet (2015) 11(3):e1005079. 10.1371/journal.pgen.1005079 25785854 PMC4364777

[55] Ribas V Nguyen MTA Henstridge DC Nguyen A-K Beaven SW Watt MJ. Impaired Oxidative Metabolism and Inflammation are Associated With Insulin Resistance in ERalpha-deficient Mice. Am J Physiol Endocrinol Metab (2010) 298(2):E304–19. 10.1152/ajpendo.00504.2009 PMC2822483 19920214

[56] Zhu L Shi J Luu TN Neuman JC Trefts E Yu S. Hepatocyte Estrogen Receptor Alpha Mediates Estrogen Action to Promote Reverse Cholesterol Transport During Western-Type Diet Feeding. Mol Metab (2018) 8:106–16. 10.1016/j.molmet.2017.12.012 PMC5985047 29331506

[57] Badeau RM Metso J Wähälä K Tikkanen MJ Jauhiainen M. Human Macrophage Cholesterol Efflux Potential is Enhanced by HDL-Associated 17beta-Estradiol Fatty Acyl Esters. J Steroid Biochem Mol Biol (2009) 116(1-2):44–9. 10.1016/j.jsbmb.2009.04.008 19406243

[58] Villa A Della Torre S Stell A Cook J Brown M Maggi A. Tetradian Oscillation of Estrogen Receptor α Is Necessary to Prevent Liver Lipid Deposition. Proc Natl Acad Sci USA (2012) 109(29):11806–11. 10.1073/pnas.1205797109 PMC3406808 22761311

[59] Zhang Y Klein K Sugathan A Nassery N Dombkowski A Zanger UM. Transcriptional Profiling of Human Liver Identifies Sex-Biased Genes Associated With Polygenic Dyslipidemia and Coronary Artery Disease. PloS One (2011) 6(8):e23506. 10.1371/journal.pone.0023506 21858147 PMC3155567

[60] Penniman CM Suarez Beltran PA Bhardwaj G Junck TL Jena J Poro K. Loss of FoxOs in Muscle Reveals Sex-Based Differences in Insulin Sensitivity But Mitigates Diet-Induced Obesity. Mol Metab (2019) 30:203–20. 10.1016/j.molmet.2019.10.001 PMC6819874 31767172

[61] Fatima LA Campello RS Barreto-Andrade JN Passarelli M Santos RS Clegg DJ. Estradiol Stimulates Adipogenesis and Slc2a4/GLUT4 Expression *Via* ESR1-Mediated Activation of CEBPA. Mol Cell Endocrinol (2019) 498:110447. 10.1016/j.mce.2019.05.006 31100494

[62] Alejandro EU. Males Require Estrogen Signaling Too: Sexual Dimorphism in the Regulation of Glucose Homeostasis by Nuclear Erα. Diabetes (2019) 68(3):471–3. 10.2337/dbi18-0046 PMC6385747 30787067

[63] Tsai S-Y Rodriguez AA Dastidar SG Del Greco E Carr KL Sitzmann JM. Increased 4E-BP1 Expression Protects Against Diet-Induced Obesity and Insulin Resistance in Male Mice. Cell Rep (2016) 16(7):1903–14. 10.1016/j.celrep.2016.07.029 PMC4988876 27498874

[64] Velasco M Ortiz-Huidobro RI Larqué C Sánchez-Zamora YI Romo-Yáñez J Hiriart M. Sexual Dimorphism in Insulin Resistance in a Metabolic Syndrome Rat Model. Endocr Connect (2020) 9(9):890–902. 10.1530/EC-20-0288 33069157 PMC7583132

[65] Pettersson US Waldén TB Carlsson P-O Jansson L Phillipson M. Female Mice are Protected Against High-Fat Diet Induced Metabolic Syndrome and Increase the Regulatory T Cell Population in Adipose Tissue. PloS One (2012) 7(9):e46057. 10.1371/journal.pone.0046057 23049932 PMC3458106

[66] Gambineri A. Sex Hormones, Obesity and Type 2 Diabetes-is There a Link? EJEA (2018) 8(1):R1–R9. 10.1530/endoabs.56.S18.1 PMC6320346 30533003

[67] Zitzmann M. Testosterone Deficiency, Insulin Resistance and the Metabolic Syndrome. Nat Rev Endocrinol (2009) 5(12):673–81. 10.1038/nrendo.2009.212 19859074

[68] Navarro G Allard C Xu W Mauvais-Jarvis F. The Role of Androgens in Metabolism, Obesity, and Diabetes in Males and Females. Obes (Silver Spring) (2015) 23(4):713–9. 10.1002/oby.21033 PMC4380643 25755205

[69] Rubin KH Glintborg D Nybo M Abrahamsen B Andersen M. Development and Risk Factors of Type 2 Diabetes in a Nationwide Population of Women With Polycystic Ovary Syndrome. J Clin Endocrinol Metab (2017) 102(10):3848–57. 10.1210/jc.2017-01354 28938447

[70] Xu W Schiffer L Qadir MMF Zhang Y Hawley J Mota De Sa P. Intracrine Testosterone Activation in Human Pancreatic β-Cells Stimulates Insulin Secretion. Diabetes (2020) 69(11):2392–9. 10.2337/db20-0228 PMC7576567 32855171

[71] Mauvais-Jarvis F Clegg DJ Hevener AL. The Role of Estrogens in Control of Energy Balance and Glucose Homeostasis. Endocr Rev (2013) 34(3):309–38. 10.1210/er.2012-1055 PMC3660717 23460719

[72] Tiano J Mauvais-Jarvis F. Selective Estrogen Receptor Modulation in Pancreatic β-Cells and the Prevention of Type 2 Diabetes. Islets (2012) 4(2):173–6. 10.4161/isl.19747 PMC3396704 22543247

[73] Vital P Larrieta E Hiriart M. Sexual Dimorphism in Insulin Sensitivity and Susceptibility to Develop Diabetes in Rats. J Endocrinol (2006) 190(2):425–32. 10.1677/joe.1.06596 16899575

[74] Basu A Dube S Basu R. Men Are From Mars, Women Are From Venus: Sex Differences in Insulin Action and Secretion. Adv Exp Med Biol (2017) 1043:53–64. 10.1007/978-3-319-70178-3_4 29224090

[75] Morimoto S Mendoza-Rodríguez CA Hiriart M Larrieta ME Vital P Cerbón MA. Protective Effect of Testosterone on Early Apoptotic Damage Induced by Streptozotocin in Rat Pancreas. J Endocrinol (2005) 187(2):217–24. 10.1677/joe.1.06357 16293769

[76] Navarro G Xu W Jacobson DA Wicksteed B Allard C Zhang G. Extranuclear Actions of the Androgen Receptor Enhance Glucose-Stimulated Insulin Secretion in the Male. Cell Metab (2016) 23(5):837–51. 10.1016/j.cmet.2016.03.015 PMC4864089 27133133

[77] Mishra JS More AS Kumar S. Elevated Androgen Levels Induce Hyperinsulinemia Through Increase in Ins1 Transcription in Pancreatic Beta Cells in Female Rats. Biol Reprod (2018) 98(4):520–31. 10.1093/biolre/ioy017 PMC6279097 29365042

[78] Velasco M Díaz-García CM Larqué C Hiriart M. Modulation of Ionic Channels and Insulin Secretion by Drugs and Hormones in Pancreatic Beta Cells. Mol Pharmacol (2016) 90(3):341–57. 10.1124/mol.116.103861 27436126

[79] Wong WPS Tiano JP Liu S Hewitt SC Le May C Dalle S. Extranuclear Estrogen Receptor-Alpha Stimulates Neurod1 Binding to the Insulin Promoter and Favors Insulin Synthesis. Proc Natl Acad Sci USA (2010) 107(29):13057–62. 10.1073/pnas.0914501107 PMC2919966 20616010

[80] Sharma G Prossnitz ER. Mechanisms of Estradiol-Induced Insulin Secretion by the G Protein-Coupled Estrogen Receptor GPR30/GPER in Pancreatic Beta-Cells. Endocrinology (2011) 152(8):3030–9. 10.1210/en.2011-0091 PMC3138237 21673097

[81] Heart Disease and Stroke Statistics. Update - American College of Cardiology (2017). Available at: https://www.acc.org/latest-in-cardiology/ten-points-to-remember/2017/02/09/14/58/heart-disease-and-stroke-statistics-2017.

[82] Smith GI Mittendorfer B Klein S. Metabolically Healthy Obesity: Facts and Fantasies. J Clin Invest (2019) 129(10):3978–89. 10.1172/JCI129186 PMC6763224 31524630

[83] Salaün H Thariat J Vignot M Merrouche Y Vignot S. Obesity and Cancer. Bull Cancer (2017) 104(1):30–41. 10.1016/j.bulcan.2016.11.012 28007295

[84] Chang E Varghese M Singer K. Gender and Sex Differences in Adipose Tissue. Curr Diabetes Rep (2018) 18(9):69. 10.1007/s11892-018-1031-3 PMC6525964 30058013

[85] Geer EB Shen W. Gender Differences in Insulin Resistance, Body Composition, and Energy Balance. Gend Med (2009) 6 Suppl 1:60–75. 10.1016/j.genm.2009.02.002 19318219 PMC2908522

[86] Attie AD Scherer PE. Adipocyte Metabolism and Obesity. J Lipid Res (2009) 50 Suppl:S395–9. 10.1194/jlr.R800057-JLR200 PMC2674728 19017614

[87] Perreault L Bergman BC Hunerdosse DM Eckel RH. Altered Intramuscular Lipid Metabolism Relates to Diminished Insulin Action in Men, But Not Women, in Progression to Diabetes. Obes (Silver Spring) (2010) 18(11):2093–100. 10.1038/oby.2010.76 PMC3230250 20379150

[88] Garcia-Carrizo F Priego T Szostaczuk N Palou A Picó C. Sexual Dimorphism in the Age-Induced Insulin Resistance, Liver Steatosis, and Adipose Tissue Function in Rats. Front Physiol (2017) 8:445. 10.3389/fphys.2017.00445 28744221 PMC5504177

[89] Bhagwandin C Ashbeck EL Whalen M Bandola-Simon J Roche PA Szajman A. The E3 Ubiquitin Ligase March1 Regulates Glucose-Tolerance and Lipid Storage in a Sex-Specific Manner. PloS One (2018) 13(10):e0204898. 10.1371/journal.pone.0204898 30356278 PMC6200199

[90] Moresi V Adamo S Berghella L. The JAK/STAT Pathway in Skeletal Muscle Pathophysiology. Front Physiol (2019) 10:500. 10.3389/fphys.2019.00500 31114509 PMC6502894

[91] Asai K Hiki N Mimura Y Ogawa T Unou K Kaminishi M. Gender Differences in Cytokine Secretion by Human Peripheral Blood Mononuclear Cells: Role of Estrogen in Modulating LPS-Induced Cytokine Secretion in an Ex Vivo Septic Model. Shock (2001) 16(5):340–3. 10.1097/00024382-200116050-00003 11699070

[92] Aomatsu M Kato T Kasahara E Kitagawa S. Gender Difference in Tumor Necrosis Factor-α Production in Human Neutrophils Stimulated by Lipopolysaccharide and Interferon-γ. Biochem Biophys Res Commun (2013) 441(1):220–5. 10.1016/j.bbrc.2013.10.042 24140406

[93] Ghisletti S Meda C Maggi A Vegeto E. 17beta-Estradiol Inhibits Inflammatory Gene Expression by Controlling NF-Kappab Intracellular Localization. Mol Cell Biol (2005) 25(8):2957–68. 10.1128/MCB.25.8.2957-2968.2005 PMC1069609 15798185

[94] Nolan CJ Prentki M. Insulin Resistance and Insulin Hypersecretion in the Metabolic Syndrome and Type 2 Diabetes: Time for a Conceptual Framework Shift. Diabetes Vasc Dis Res (2019) 16(2):118–27. 10.1177/1479164119827611 30770030

[95] Larqué C Velasco M Navarro-Tableros V Duhne M Aguirre J Gutiérrez-Reyes G. Early Endocrine and Molecular Changes in Metabolic Syndrome Models. IUBMB Life (2011) 63(10):831–9. 10.1002/iub.544 21905198

[96] Aguayo-Mazzucato C Sanchez-Soto C Godinez-Puig V Gutiérrez-Ospina G Hiriart M. Restructuring of Pancreatic Islets and Insulin Secretion in a Postnatal Critical Window. PloS One (2006) 1:e35. 10.1371/journal.pone.0000035 17183663 PMC1762382

[97] Høeg LD Sjøberg KA Jeppesen J Jensen TE Frøsig C Birk JB. Lipid-Induced Insulin Resistance Affects Women Less Than Men and Is Not Accompanied by Inflammation or Impaired Proximal Insulin Signaling. Diabetes (2011) 60(1):64–73. 10.2337/db10-0698 20956497 PMC3012198

[98] Gupte AA Pownall HJ Hamilton DJ. Estrogen: An Emerging Regulator of Insulin Action and Mitochondrial Function. J Diabetes Res (2015) 2015:916585. 10.1155/2015/916585 25883987 PMC4391691

[99] Amengual-Cladera E Lladó I Gianotti M Proenza AM. Sex Differences in the Effect of High-Fat Diet Feeding on Rat White Adipose Tissue Mitochondrial Function and Insulin Sensitivity. Metab Clin Exp (2012) 61(8):1108–17. 10.1016/j.metabol.2011.12.016 22401878

[100] Van Sinderen M Steinberg G Jorgensen SB Honeyman J Chow JDY Simpson ER. Sexual Dimorphism in the Glucose Homeostasis Phenotype of the Aromatase Knockout (ArKO) Mice. J Steroid Biochem Mol Biol (2017) 170:39–48. 10.1016/j.jsbmb.2016.05.013 27353462

[101] Méndez-García LA Trejo-Millán F Martínez-Reyes CP Manjarrez-Reyna AN Esquivel-Velázquez M Melendez-Mier G. Infliximab Ameliorates Tumor Necrosis Factor-Alpha-Induced Insulin Resistance by Attenuating Ptp1b Activation in 3T3L1 Adipocytes In Vitro. Scand J Immunol (2018) 88(5):e12716. 10.1016/j.jsbmb.2016.05.013 30260514

[102] Camporez JP Lyu K Goldberg EL Zhang D Cline GW Jurczak MJ. Anti-Inflammatory Effects of Oestrogen Mediate the Sexual Dimorphic Response to Lipid-Induced Insulin Resistance. J Physiol (Lond) (2019) 597(15):3885–903. 10.1113/JP277270 PMC6876753 31206703

[103] Garcia-Barrado MJ Iglesias-Osma MC Moreno-Viedma V Pastor Mansilla MF Gonzalez SS Carretero J. Differential Sensitivity to Adrenergic Stimulation Underlies the Sexual Dimorphism in the Development of Diabetes Caused by Irs-2 Deficiency. Biochem Pharmacol (2011) 81(2):279–88. 10.1016/j.bcp.2010.10.008 20959116

[104] Kubota N Terauchi Y Tobe K Yano W Suzuki R Ueki K. Insulin Receptor Substrate 2 Plays a Crucial Role in Beta Cells and the Hypothalamus. J Clin Invest (2004) 114(7):917–27. 10.1172/JCI21484 PMC518663 15467830

[105] Laplante M Sabatini DM. Mtor Signaling in Growth Control and Disease. Cell (2012) 149(2):274–93. 10.1016/j.cell.2012.03.017 PMC3331679 22500797

[106] Um SH Frigerio F Watanabe M Picard F Joaquin M Sticker M. Absence of S6K1 Protects Against Age- and Diet-Induced Obesity While Enhancing Insulin Sensitivity. Nature (2004) 431(7005):200–5. 10.1038/nature02866 15306821

[107] Jaldin-Fincati JR Pavarotti M Frendo-Cumbo S Bilan PJ Klip A. Update on GLUT4 Vesicle Traffic: A Cornerstone of Insulin Action. Trends Endocrinol Metab (2017) 28(8):597–611. 10.1016/j.tem.2017.05.002 28602209

[108] Biessels GJ Reagan LP. Hippocampal Insulin Resistance and Cognitive Dysfunction. Nat Rev Neurosci (2015) 16(11):660–71. 10.1038/nrn4019 26462756

[109] Campello RS Fátima LA Barreto-Andrade JN Lucas TF Mori RC Porto CS. Estradiol-Induced Regulation of GLUT4 in 3T3-L1 Cells: Involvement of ESR1 and AKT Activation. J Mol Endocrinol (2017) 59(3):257–68. 10.1530/JME-17-0041 28729437

